# Step-by-step technique for MIS robotic spinal fusion surgery

**DOI:** 10.3171/2025.4.FOCVID258

**Published:** 2025-07-01

**Authors:** Giuseppe Di Nuzzo, Francesco Corrivetti, Vincenzo Seneca, Carmela Chiaramonte, Roberto Granata, Matteo de Notaris, Giuseppe Catapano

**Affiliations:** 1 Department of Neurosurgery, Ospedale del Mare, Naples, Italy; and; 2 Unit of Neurosurgery, University Hospital San Giovanni di Dio e Ruggi d’Aragona, University of Salerno, Salerno, Italy

**Keywords:** robotic surgery, minimally invasive surgery, spinal fusion, robotics

## Abstract

The clinical use of new technologies has the goal to produce healthcare advancements. In this context, robotic assistance for spinal surgery carries several potential benefits, including improved accuracy, precision, and efficiency, and such technology is making its way into neurosurgical operating rooms with increasing frequency.

However, shifting the spinal fusion technique from the traditional freehand C-arm–guided technique to a robot-assisted technique can be challenging. In this video, the authors present the step-by-step robot-assisted technique, using the Mazor X Stealth robotic technology, as performed in their department for lumbar pedicle screw placement.

The video can be found here: https://stream.cadmore.media/r10.3171/2025.4.FOCVID258

## Transcript

### 0:21

In this video, we present our step-by-step technique for robotic spinal fusion surgery.

### 0:31

Pedicle screw fixation technique has gradually become a routine operation in spinal surgery. Complications can occur and have catastrophic consequences for the patient, potentially impacting the functionality of the spine and stability of the vertebrae. Mismanagement in implant placement can result in deformities, instabilities, and risk of nerve and vascular damage.

### 0:54

Moreover, freehand technique of pedicle screw implant has major limitations regarding accuracy, radiation exposure, invasiveness, and efficiency.

### 1:01

And those are the reasons why robotic surgery, that is a widely adopted healthcare advancement, has been applied to spinal pathology. And this technique uses both spinal navigation and provides robotic assistance and can be divided in four fundamental steps.

### 1:26

The first phase is the preoperative planning, in which we are able to design the trajectory of the screws, but moreover, and this is a critical advantage, to design the position of the rods in order to be able to make rods corresponding to the screws’ tulip, obtaining the desired degree of lumbar lordosis correction. And moreover, it’s worth noting that this phase can be performed not only preoperatively, but also several hours before surgery.

### 2:01

The second step is the calibration of the robotic arm and the scanning of the operative field.

### 2:10

Then the operative phase, implanting the screws and the robotic assistance and spinal navigation.

### 2:18

And the last phase, that is the confirmation of the screw placement.

### 2:24

And these critical steps of robotic-assisted spinal screw implants are illustrated in this clinical case performed at the Ospedale del Mare Hospital in Naples of a 52-year-old woman that accidentally fell at home. And performed a CT scan that showed an incomplete burst fracture of D12. And radiological examination show very narrow pedicle at D11 and D12.

### 3:01

Therefore, she underwent percutaneous stabilization using the robotic-assisted procedure. In this preoperative planning, we selected the region of interest from the preoperative CT scan, identifying the three levels (D11, D12, and L1) in which we will perform the stabilization and the screw implant. And then proceeding with the preoperative plan, designing the dimension of the screws and the trajectory inside the vertebrae, but moreover designing the positioning of the rods, verifying the trajectory of the screw inside the pedicle, and then rendering and showing a 3D reconstruction of our construct together with the rods.

### 4:06

The previously described procedure was performed at the Ospedale del Mare Hospital in Naples.

### 4:25

At first, the robotic arm needs to be calibrated. The robotic system needs to verify accuracy. Then the C-arm needs to be calibrated to fuse the radiological images performed preoperatively with the intraoperative x-rays.

### 4:46

The C-arm calibration. We perform the fixation of the robot to the operative table. We use a plate that attaches itself to the operative table and then allows the fixation of the robot to the operative table.

### 5:10

This is the instrument setting that is used to perform the procedure—two pins and one bridge for patient fixation to the robotic arm. The robotic tools need to be attached to the arm.

### 5:29

At first, a neuronavigation reference has to be attached to the robotic arm. And then this tool is a system for patient hookup that allows to construct a bridge with the robot and the patient, positioning two pins on the cranial and the caudal spinal process, and bridge between these two pins that is later attached to the robotic tool.

### 6:12

In this way, the patient, the table, and the robot are attached altogether.

### 6:23

The next step is the operative field scanning by the robotic arm, followed by the neuronavigation reference fixation and calibration.

### 6:35

And then our x images are acquired and fused with the preoperative CT scan.

### 6:45

The preoperative planning is confirmed. And then the operative phase is started.

### 6:54

The robotic arm gets in the position of the implant of the first screw, proceeding with a robotic-assisted skin incision, followed by a tissue dilator insertion. That will be the guidance for the neuronavigated drill that allows to perform a drilling inside the pedicle to the vertebra. And then followed by the implant of the screw.

### 7:42

After the first screw implantation, the robotic arm is positioned for the next procedure that is repeated for each screw implant. Rod placement, not shown in this video, is then performed using the same technique of standard percutaneous fixation.

### 8:09

Postoperative period was uneventful. The patient ambulate independently without neurological deficits at day 1, obtaining a good cosmetic result provided by the small skin incision obtained by the minimally invasive robotic technique.

### 8:26

Postoperative CT scan examination showed the correct positioning of the screw, and the correct execution of the spinal fusion and moreover the exact correspondence of the implanted screw with the preoperative plan.

### 8:49

Concluding, robot-assisted spinal stabilization can improve safety and efficiency of the procedure. Screw accuracy due to the absence of physiological factors allows for a millimeter precision, which is unachievable even by the best surgeons. Moreover, robotic surgery carries huge potential for future improvement of this technology.

### 9:09 References^1–5^

## Disclosures

The authors report no conflict of interest concerning the materials or methods used in this study or the findings specified in this publication.

## Author Contributions

Primary surgeon: Di Nuzzo, Chiaramonte, Granata, Catapano. Assistant surgeon: Seneca. Editing and drafting the video and abstract: Corrivetti, Di Nuzzo, Granata, de Notaris. Critically revising the work: Corrivetti, Di Nuzzo, Seneca, Granata. Reviewed submitted version of the work: Corrivetti, Di Nuzzo. Approved the final version of the work on behalf of all authors: Corrivetti. Supervision: Corrivetti, Di Nuzzo, Granata.

## Supplemental Information

### Patient Informed Consent

The necessary patient informed consent was obtained in this study.

## References

[1] O’Connor TE O’Hehir MM Khan A Mazor X Stealth robotic technology: a technical note World Neurosurg 2021 145 435 442 33059080 10.1016/j.wneu.2020.10.010

[2] Zhao W Wang Y Zhang H Analysis of the screw accuracy and postoperative efficacy of screw placement in single position and bipedal position in robot-assisted oblique lumbar interbody fusion: preliminary results of Mazor X Stealth usage Orthop Surg 2024 16 2 401 411 38151861 10.1111/os.13972 PMC10834206

[3] Lee NJ Zuckerman SL Buchanan IA Is there a difference between navigated and non-navigated robot cohorts in robot-assisted spine surgery? A multicenter, propensity-matched analysis of 2,800 screws and 372 patients Spine J 2021 21 9 1504 1512 34022461 10.1016/j.spinee.2021.05.015

[4] Mason A Paulsen R Babuska JM The accuracy of pedicle screw placement using intraoperative image guidance systems J Neurosurg Spine 2014 20 2 196 203 24358998 10.3171/2013.11.SPINE13413

[5] Perfetti DC Kisinde S Rogers-LaVanne MP Satin AM Lieberman IH Robotic spine surgery: past, present, and future Spine (Phila Pa 1976) 2022 47 13 909 921 35472043 10.1097/BRS.0000000000004357

